# scLAP: A Liver-Adapted Contrastive Shared-Latent Framework for Pseudo-Pairing Single-Cell Transcriptomes and Chromatin Accessibility Profiles

**DOI:** 10.3390/ijms27219888

**Published:** 2026-11-04

**Authors:** Hongfei Li, Jiechen Wang, Huan Yang

**Affiliations:** 1 Yangtze Delta Region Institute (Quzhou), University of Electronic Science and Technology of China, Quzhou 324003, China; lihongfei@nefu.edu.cn (H.L.); wangjc@nefu.edu.cn (J.W.); 2 Institute of Fundamental and Frontier Sciences, University of Electronic Science and Technology of China, Chengdu 610054, China

**Keywords:** single-cell multi-omics, hepatocellular carcinoma, contrastive learning, chromatin accessibility, pseudo-pairing

## Abstract

Matched single-cell RNA sequencing (scRNA-seq) and single-cell assay for transposase-accessible chromatin sequencing (scATAC-seq) profiles from the same cells remain limited in hepatocellular carcinoma (HCC), hindering cell-resolved integration of transcriptional programs and regulatory chromatin states. To address this challenge, we developed single-cell Liver-adapted Alignment and Pseudo-pairing (scLAP), a liver-adapted contrastive shared-latent framework for cross-study RNA-ATAC pseudo-pairing. scLAP was trained on a paired liver single-nucleus multiome reference using 2000 highly variable RNA features and 697 ATAC features, including 647 transcription factor motif activity scores and 50 latent semantic indexing components. On a prespecified cell-level test partition (*n* = 3235), scLAP achieved an ATAC-to-RNA Top 10 exact-pair retrieval rate of 21.48% and a mean fraction of samples closer than the true match (FOSCTTM) of 0.0431. In six leave-one-sample-out folds, the corresponding macro means were 5.23% and 0.2278, respectively, indicating above-random ranking but limited exact-pair transfer to unseen samples. A validation-selected scButterfly configuration using 20,000 native ATAC peaks achieved 8.93% Top 10 retrieval and a mean FOSCTTM of 0.0811 on the same test cells; comparisons with Con-AAE and scMODAL remained study-specific adaptations. Applying reciprocal retrieval, a validation-derived cosine-similarity threshold, and greedy one-to-one assignment to independent HCC datasets yielded 4007 similarity-filtered candidate RNA-ATAC pseudo-pairs. Downstream analyses identified an NRCAM-associated accessibility candidate and separate exploratory MET-, CAV1-, and AP-1-associated observations.

## 1. Introduction

Primary liver cancer (PLC) represents a major and increasing global health burden and remains one of the leading causes of cancer-related mortality worldwide [1,2]. Hepatocellular carcinoma (HCC), which accounts for approximately 90% of PLC cases, typically arises in the setting of chronic liver injury driven by viral hepatitis, alcohol-associated liver disease, or metabolic dysfunction-associated steatotic liver disease [3]. HCC progression is shaped by the accumulation of genetic and epigenetic alterations, including recurrent changes affecting the TERT promoter, TP53, and CTNNB1, as well as complex interactions among malignant cells, stromal compartments, and immune populations [3,4]. Resolving these interactions at cellular resolution is therefore essential for understanding tumor evolution and identifying potential therapeutic vulnerabilities.

Single-cell RNA sequencing (scRNA-seq) has substantially advanced the characterization of the cellular diversity of liver cancer [5,6,7]. Ma et al. demonstrated that transcriptional heterogeneity among tumor cells is associated with microenvironmental remodeling and adverse clinical features [8]. Single-cell profiling has also revealed the complexity of the HCC immune microenvironment [9], connected transcriptional states with tumor evolution and clonal dynamics [10], and identified spatially conserved ligand–receptor interactions between malignant and immune cells across tumor regions [11]. These findings highlight that HCC cannot be fully described by bulk-level measurements. However, transcriptomic profiles represent an important but incomplete layer of tumor biology: they capture cellular states at the level of gene expression more directly than the cis-regulatory chromatin programs that may precede or sustain them.

Integrating transcriptomic and chromatin accessibility profiles offers an opportunity to connect gene-expression changes with their underlying regulatory mechanisms [12,13,14]. Single-cell multiomic assays can associate accessible chromatin regions, including distal regulatory elements, with transcriptional variation and cell-state trajectories [15]. Paired liver single-cell multiome analysis has further demonstrated the value of combining RNA and chromatin accessibility information for dissecting disease-associated regulatory programs [16]. However, matched RNA–ATAC measurements remain limited in many clinical cohorts, including PLC, whereas independently generated scRNA-seq and single-cell assay for transposase-accessible chromatin sequencing (scATAC-seq) datasets are increasingly available. This creates a practical need for computational strategies that can recover traceable RNA–ATAC cell pairings across independently generated datasets.

Deep-learning approaches have provided powerful frameworks for cross-modality integration [17,18,19]. scButterfly uses dual-aligned variational autoencoders for cross-modality translation in single-cell data [20]. scMODAL integrates multiple modalities through shared latent representations while incorporating prior feature relationships, including links between gene expression and gene activity, for RNA–ATAC alignment [21]. Con-AAE combines adversarial autoencoders, cycle consistency, and contrastive learning to improve single-cell multiomics integration [22]. These approaches establish important foundations for cross-modality analysis, but their primary objectives differ from the problem of cross-study RNA–ATAC pseudo-pairing. Their direct transfer is not trivial: cross-modality translation and one-to-one cell retrieval represent related but distinct objectives; gene activity summaries used for linked-feature construction may incompletely capture distal enhancer information; annotations that facilitate contrastive integration may be inconsistent or unavailable across clinical datasets; and peak spaces can vary substantially between studies. These challenges reflect domain-transfer issues rather than universal limitations of the original frameworks.

**Figure 1:**
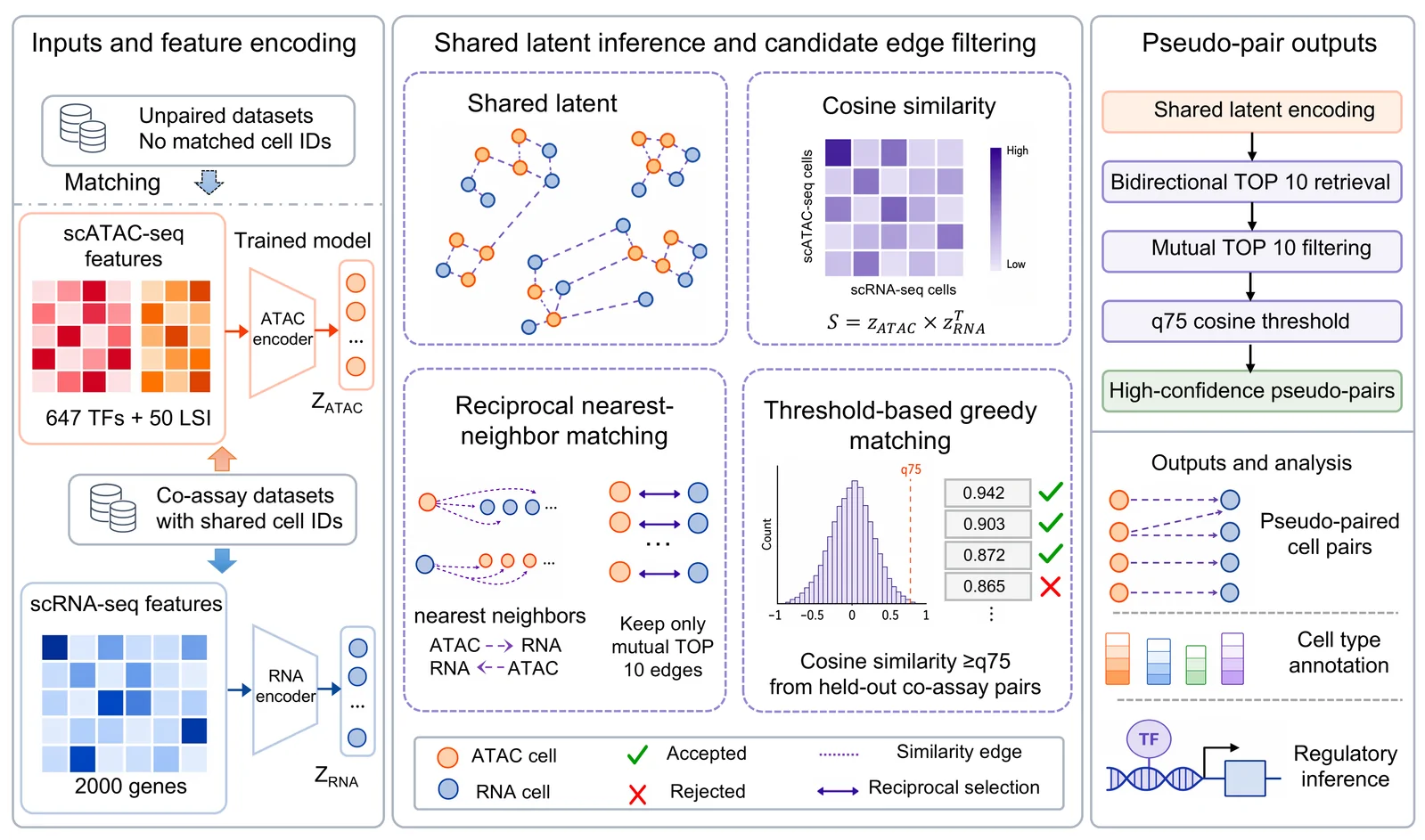
Overview of scLAP feature construction and cross-study RNA-ATAC pseudo-pairing. scATAC-seq profiles are represented by 647 transcription factor motif-activity features and 50 LSI components, whereas scRNA-seq profiles are represented by 2000 highly variable genes selected from the paired reference. Modality-specific encoders map both inputs to L2-normalized embeddings in a shared latent space. Candidate ATAC-RNA edges are generated by bidirectional Top 10 retrieval, reciprocal selection, and a q75 cosine-similarity threshold derived from true-pair similarities in the co-assay validation partition, followed by greedy one-to-one assignment. The resulting similarity-filtered candidates support exploratory cell-composition and regulatory analyses. TF, transcription factor; LSI, latent semantic indexing.

Here, we developed single-cell Liver-adapted Alignment and Pseudo-pairing (scLAP), a contrastive shared-latent learning framework for constructing model-guided RNA–ATAC pseudo-pair candidates across liver datasets (Figure 1). scLAP learns cell correspondences from a liver single-cell multiome reference and integrates liver-relevant RNA features with ATAC-derived transcription factor motif activity and latent semantic indexing (LSI) features. The framework preserves modality translation and cell-level retrieval structure, using similarity-filtered reciprocal matching followed by one-to-one assignment in unpaired cohorts. Applied to independent HCC datasets, scLAP generated candidate RNA–ATAC pseudo-pairs for exploratory chromatin analysis, including an NRCAM-associated accessibility candidate and separate MET-, CAV1-, and AP-1-associated observations requiring further validation.

## 2. Results

### 2.1. scLAP Preserved Paired-Cell Retrieval Under Prespecified Cell-Level Partitions and Study-Specific Baseline Adaptations

We first evaluated whether scLAP preserved paired-cell identity in the shared latent space rather than merely reconstructing an averaged RNA profile. On the prespecified paired-liver cell-level test partition (n = 3235), scLAP achieved an ATAC-to-RNA Top 10 exact-pair retrieval rate of 21.48%, a Top 1 retrieval rate of 4.82%, and a mean FOSCTTM of 0.0431. The corresponding validation results were comparable (Top 10, 21.89%; mean FOSCTTM, 0.0438), with mean cell-wise Pearson correlations of 0.6631 and 0.6650 on the test and validation partitions, respectively (Table 1). All six source samples were represented in every partition; therefore, these results quantify within-reference cell-level retrieval and do not establish generalization to unseen donors or samples. A mean FOSCTTM of 0.0431 means that, on average, 4.31% of candidate RNA cells ranked ahead of the true partner; it does not imply 95.69% exact-pair accuracy.

To assess generalization beyond the samples used for model fitting, we repeated the complete three-stage training procedure in six leave-one-sample-out folds. Each fold excluded all cells from one source sample from both training and checkpoint selection, and exact-pair retrieval was evaluated only within that unseen sample. Across the six held-out samples, macro-mean ATAC-to-RNA Top 1 and Top 10 retrieval were 0.90% (95% CI, 0.20–1.60%) and 5.23% (95% CI, 1.89–8.57%), respectively; mean FOSCTTM was 0.2278 (95% CI, 0.1718–0.2838), and mean cell-wise Pearson correlation was 0.6208 (95% CI, 0.5146–0.7270; Supplementary Table S1). Top 10 retrieval remained 7.0–20.8-fold above the sample-size-specific random expectation, but its absolute level showed that exact-cell transfer to unseen samples was limited.

We also evaluated the complete mutual Top 10, similarity-threshold, and greedy one-to-one procedure on the untouched fixed test partition after deriving the q75 threshold from the validation partition. The validation-derived threshold was 0.815683. It retained 2320 of 3235 test cells, including 106 exact pairs, corresponding to 4.57% retained-pair precision, 3.28% exact-pair recall, and 71.72% coverage; 96.12% of retained pairs nevertheless shared the same source-sample label. Raising the cutoff to the validation q95 or q99 thresholds increased exact-pair precision to 10.41% or 16.35% but reduced coverage to 16.63% or 3.21%, respectively (Supplementary Table S2). Thus, the filtering rule captured sample and cell-state proximity more strongly than exact cell identity.

**Figure 2:**
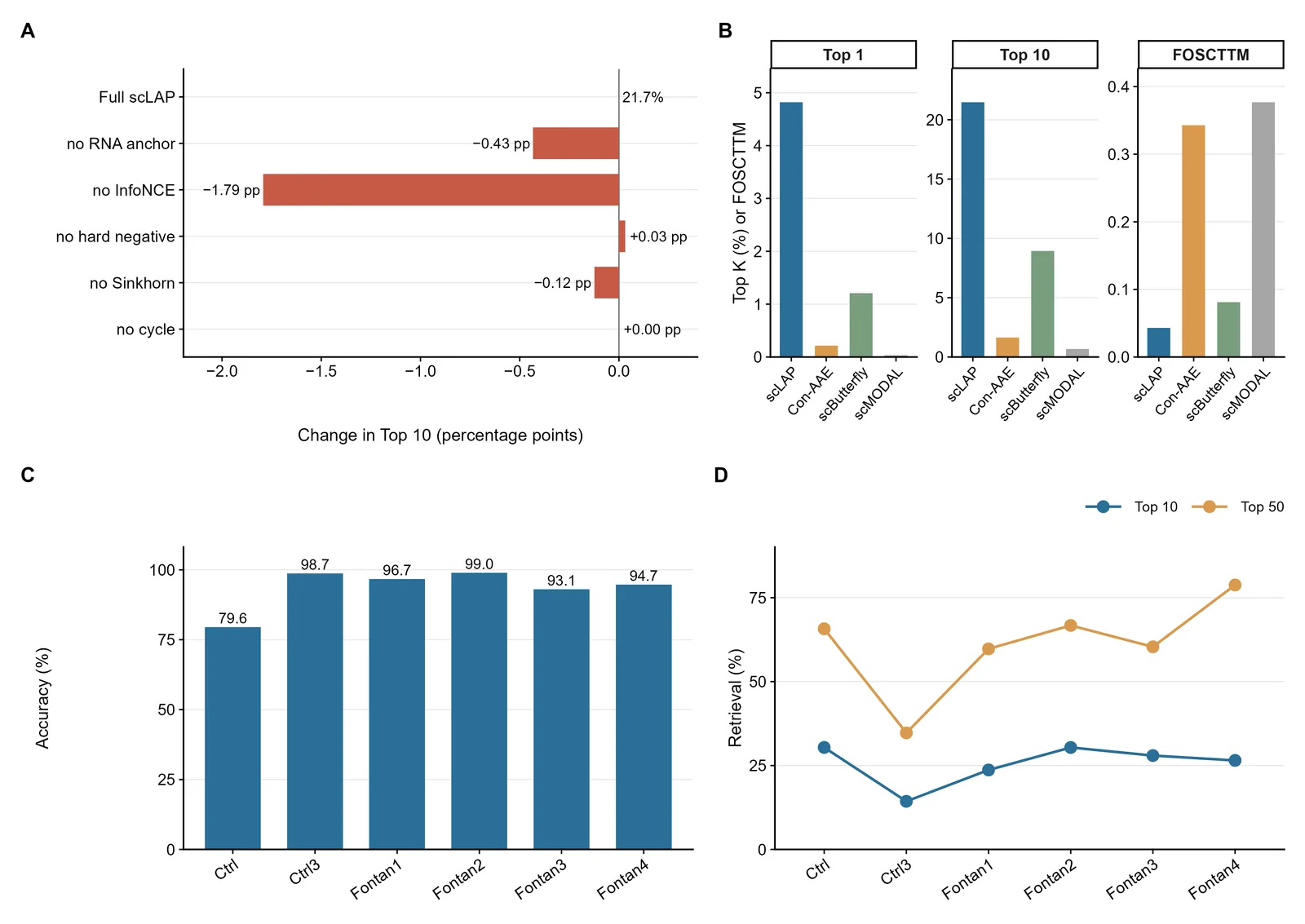
scLAP module ablation and fixed-split co-assay benchmarking. (**A**) Controlled short-run single-component ablations from a common initialization. (**B**) Fixed-split comparison on the paired-liver test partition (n = 3235). (**C**) scLAP Top 1 source-sample-label retrieval. (**D**) scLAP ATAC-to-RNA Top 10 and Top 50 exact-pair retrieval stratified by sample.

Controlled 20-epoch single-component ablations from a common initialization identified symmetric InfoNCE as the component with the largest observed effect on exact-pair retrieval in this limited setting. Removing InfoNCE reduced test-set ATAC-to-RNA Top 10 retrieval from 21.67% to 19.88%, a decrease of 1.79 percentage points (Figure 2A). Here, the RNA anchor denotes freezing the RNA encoder, RNA projection head, and RNA decoder during ATAC-side fine-tuning; removing this constraint reduced Top 10 retrieval by 0.43 percentage points. Removing Sinkhorn loss or cycle consistency produced minimal Top 10 changes. Removing the hard-negative terms changed Top 10 from 21.67% to 21.70% but reduced mutual-nearest-neighbor pairs from 1014 to 988 and true-pair MNN recall from 2.16% to 1.98%, suggesting a local effect on ambiguous matches rather than a broad effect on retrieval.

We next evaluated scLAP and comparison methods on the same paired-liver cell-level test partition. A validation-selected scButterfly model using its intended peak-based input representation (20,000 training-selected native peaks) achieved ATAC-to-RNA Top 10 retrieval of 8.93% and a mean FOSCTTM of 0.0811, compared with 21.48% and 0.0431 for scLAP (Table 1 and Figure 2B). Under the study-specific adaptations used here, Con-AAE and scMODAL achieved Top 10 retrieval of 1.64% and 0.68%, respectively. A supervised ridge ATAC-to-RNA translation control was also evaluated in predicted-expression space; its Top 10 retrieval was 3.96%, compared with 4.48% for scLAP predictions in the same space (Supplementary Table S6). These comparisons support the selected configuration on this fixed dataset but do not establish general superiority over the published methods.

### 2.2. scLAP Generated Similarity-Filtered Candidate Pseudo-Pairs in Independent Unpaired HCC Datasets

**Figure 3:**
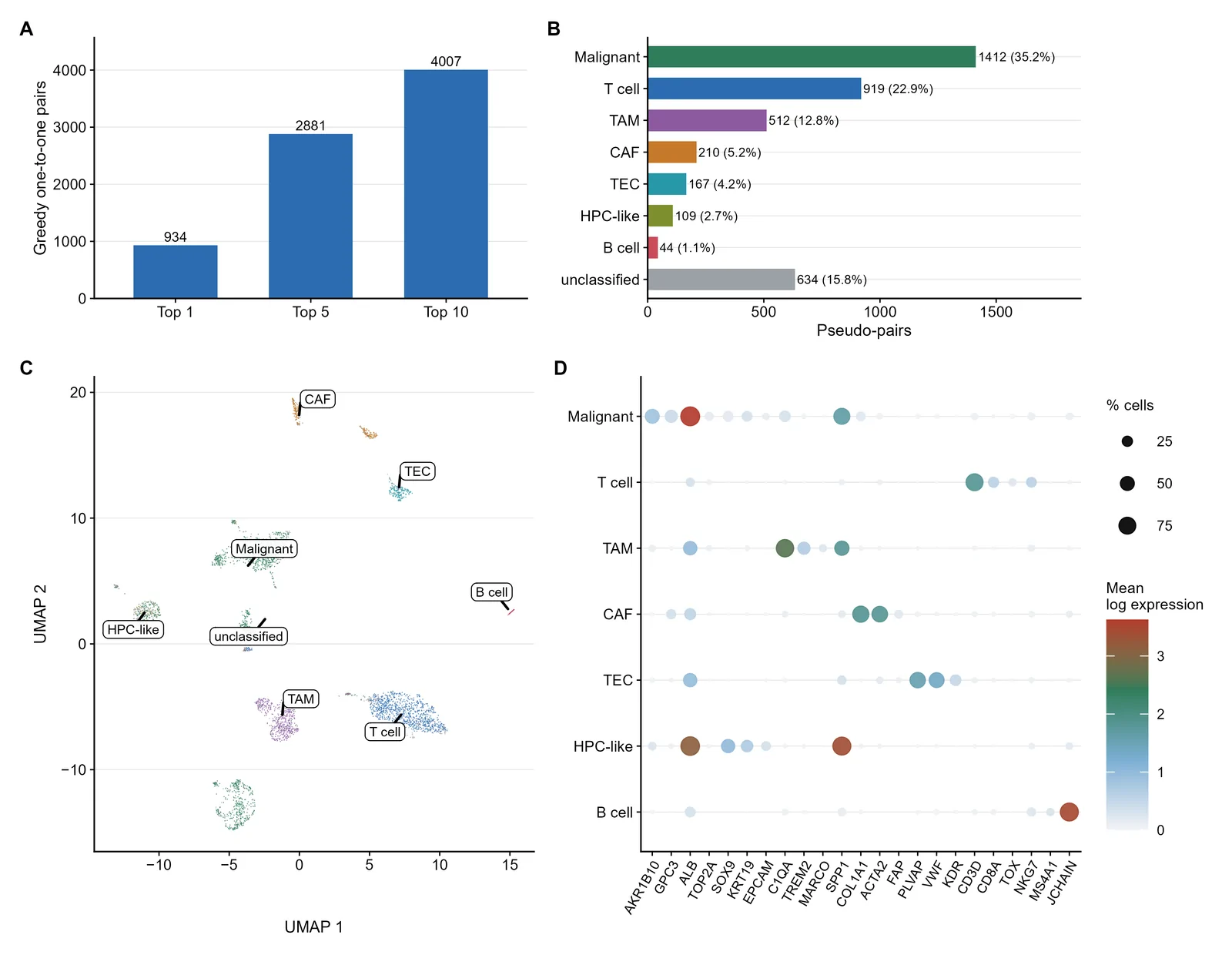
Model-guided HCC candidate pseudo-pair selection and characterization of RNA anchors. (**A**) Numbers of greedy one-to-one candidates retained by mutual Top 1, Top 5, and Top 10 retrieval using the q75 cosine-similarity threshold derived from true-pair similarities in the co-assay validation partition. (**B**) RNA-derived cellular composition of selected candidates. (**C**) Harmony-corrected UMAP visualization of selected RNA cells from the original count matrices. (**D**) Expression of canonical lineage and HCC-associated marker genes across selected RNA cell types.

We next evaluated whether the paired-liver co-assay model could generate candidate pseudo-single-cell multiomic profiles from independent, unpaired PLC datasets. After training, scLAP was frozen and applied to scRNA-seq cells from GSE125449 [8], GSE151530 [10], and GSE189903 [11], together with scATAC-seq cells from GSE227265 [23]. Mutual Top 10 retrieval in the shared latent space, a q75 cosine-similarity threshold derived from true-pair similarities in the co-assay validation partition (0.815683), and greedy one-to-one assignment yielded 4007 similarity-filtered candidate RNA-ATAC pseudo-pairs. Under the same threshold, mutual Top 1 and Top 5 matching yielded 934 and 2881 pairs, respectively (Figure 3A). The validation-derived threshold retained 4007 of the 4008 candidates obtained with the previous test-derived cutoff; the single excluded RNA anchor was unclassified and had not entered the annotated ATAC comparisons. This sensitivity analysis quantifies the coverage gained by using Top 10 but does not calibrate candidate-level correctness. The final candidates had latent cosine similarities of 0.8157–0.9446 (median, 0.8953).

The selected RNA anchors included 2514 cells from GSE151530, 1074 from GSE189903, and 419 from GSE125449, corresponding to 4.43%, 0.95%, and 4.21% of the respective input RNA cohorts. After harmonizing dataset-provided labels, the selected cells comprised 1412 malignant cells, 919 T cells, 512 tumor-associated macrophages (TAMs), 210 cancer-associated fibroblasts (CAFs), 167 tumor endothelial cells (TECs), 109 hepatic progenitor-like cells (HPC-like cells), 44 B cells, and 634 unclassified cells (Figure 3B). The composition was cohort dependent: 1195 of 1412 malignant anchors came from GSE151530, 587 of 919 T-cell anchors came from GSE189903, and all 109 HPC-like anchors came from GSE125449. Thus, multiple tumor and microenvironmental compartments were retained, but this diversity does not exclude cohort- or cell-type-dependent selection.

In a cohort-sensitivity analysis, matching each RNA cohort separately against the same ATAC pool yielded 1741 GSE125449, 3507 GSE151530, and 2101 GSE189903 candidates. Pair-identity overlap with the pooled result was 13.3%, 63.2%, and 42.6% of the respective separate-cohort sets (Supplementary Table S3), indicating that pooled candidate competition affected the cohorts unequally. ATAC barcode library 5 contributed the largest absolute fraction of candidates (22.1–23.9% across the separate matches), close to its 20.5% share of input ATAC cells. No single ATAC library dominated by absolute count, although smaller library-specific enrichments remained detectable.

To assess whether these RNA anchors retained biologically meaningful identities beyond the 2000 model-input genes, we reprocessed the original raw-count matrices for the 4007 selected RNA cells using a standard Seurat workflow. After de novo variable-gene selection, PCA, Harmony correction, and UMAP visualization, the cells separated into major tumor and microenvironmental compartments consistent with their original annotations (Figure 3C). Differential marker analysis recovered expected lineage- and state-associated markers, including ALB, GPC3, AKR1B10, AFP, KRT8/KRT18, and TOP 2A in malignant cells; SOX9, KRT19, and EPCAM in HPC-like cells; C1QA/B/C, MARCO, TREM2, SPP1, and APOE in TAMs; COL1A1/2, ACTA2, and FAP in CAFs; PLVAP, VWF, KDR, and PECAM1 in TECs; CD3D, CD8A, NKG7, IL7R, and TOX in T cells; and MS4A1, JCHAIN, and immunoglobulin-related genes in B cells (Figure 3D). These marker profiles were consistent with previously reported liver cancer cell states and marker programs, including GPC3/AFP-positive HCC, KRT19-positive progenitor-like HCC, SPP1-positive macrophages, and PLVAP-positive tumor endothelium [24,25,26,27,28].

### 2.3. Pseudo-Paired ATAC Profiles Highlighted Cell-State Regulatory Programs

We next used the matched scATAC-seq cells to assess whether pseudo-pairing could generate interpretable chromatin profiles for RNA-defined compartments. To avoid label leakage, the consensus peak universe was constructed from all 55,421 feature-eligible ATAC cells in GSE227265 before RNA-derived label transfer. All 4007 selected ATAC cells passed ArchR quality control, and 3373 annotated cells remained after excluding unclassified cells. The final 71,862-peak universe yielded 2931 marker peaks, 3802 marker GeneScores, and 47,380 co-accessibility links with a correlation of at least 0.5.

**Figure 4:**
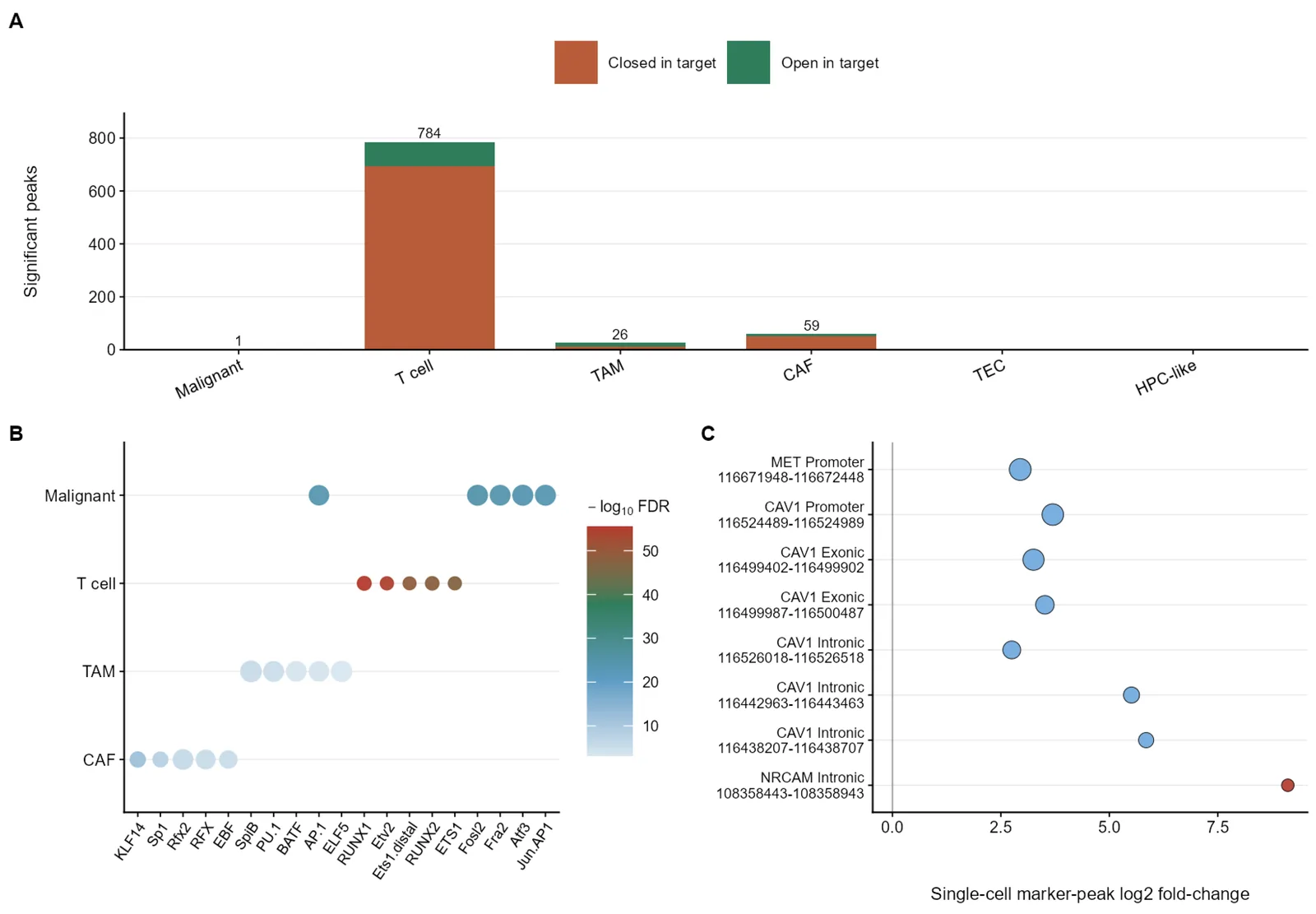
Chromatin accessibility programs associated with model-transferred RNA-defined groups. (**A**) Numbers of differentially accessible peaks from fragment-library-blocked edgeR quasi-likelihood models. (**B**) Enriched transcription factor motif families among single-cell ArchR marker peaks for malignant, T-cell, TAM, and CAF groups. Dot size denotes enrichment, and color denotes minus log10 FDR. (**C**) Evidence levels for malignant-associated candidate regions.

To reduce library-related confounding, accessibility counts were aggregated by transferred RNA cell type and fragment library, and each target group was compared with the pooled other labeled groups in the same library using edgeR quasi-likelihood generalized linear models with fragment library as a blocking factor. Significant differential peaks were detected for T-cell, CAF, TAM, and malignant groups, whereas TEC and HPC-like groups did not yield significant peaks under the prespecified thresholds (Figure 4A). Fragment libraries were treated as analysis blocks, but their equivalence to independent biological replicates was not established; inferential statements are therefore conditional on this design. Because group labels were transferred from RNA annotations, the resulting patterns are interpreted as cell-state-associated hypotheses rather than independent ATAC-based cell-type ground truth.

As an RNA-label-blind marker-concordance check, prespecified broad-lineage GeneScore panels were calculated from the ATAC data before comparison with transferred RNA labels. Among the 3373 annotated candidates, the highest-scoring ATAC marker panel agreed with the corresponding broad RNA lineage for 30.00% of cells, compared with a mean of 22.16% under 2000 fragment-library-conditioned label permutations (P = 0.00050; Supplementary Table S7). Per-lineage concordance ranged from 25.0% to 40.1%. This result supports above-null aggregate lineage structure but does not validate malignant identity or exact-cell correspondence.

Motif enrichment of marker peaks further supported biologically plausible regulatory programs. Malignant-cell peaks were enriched for AP-1-associated motifs, including FOSL2, FRA2, ATF3, AP-1, and JUN/AP1; T-cell peaks were enriched for RUNX and ETS-family motifs; TAM peaks showed enrichment for SPIB and PU.1 motifs; and CAF peaks showed enrichment for KLF14, SP1, and RFX motifs (Figure 4B). These patterns are consistent with previous PLC scATAC-seq evidence showing that transcription factor motif activity distinguishes malignant and microenvironmental states, as well as experimental evidence linking AP-1 dimers to c-Myc-associated liver cancer programs [29].

In malignant-labeled cells, exploratory ArchR marker-peak analysis identified accessible regions near MET and CAV1, including a MET promoter peak and multiple CAV1 promoter, exonic, and intronic peaks (Figure 4C). Co-accessibility analysis detected a CAV1-proximal link between chr7:116438207–116438707 and chr7:116442963–116443463 (correlation = 0.9876). In the fragment-library-blocked pseudobulk analysis, an intronic peak nearest NRCAM at chr7:108358443–108358943 met the prespecified significance criteria (log2FC = 4.885, FDR = 0.00126). The positive log2 fold change denotes greater accessibility in the malignant-labeled pseudobulks than in the pooled other labeled groups after blocking for fragment library. This accessibility association does not establish NRCAM expression, function, or causality. The MET- and CAV1-proximal peaks and AP-1 motif enrichment represent separate exploratory observations and require independent validation before they can be interpreted as a connected regulatory axis.

Unit-level inspection showed non-zero malignant-pseudobulk counts for the NRCAM-associated peak in six of nine eligible fragment libraries. In leave-one-library-out edgeR analyses, the log2 fold change remained positive in all nine runs and ranged from 4.253 to 5.537; all runs had nominal *p* < 0.05, whereas seven of nine retained FDR < 0.05 (Supplementary Table S4). Excluding library 20 or library 5 increased the FDR to 0.824 and 0.278, respectively. The direction of the accessibility association was therefore not attributable to a single library, but its multiple-testing-adjusted significance was not uniformly robust to library exclusion.

## 3. Discussion

We developed scLAP as a liver-adapted contrastive shared-latent framework for candidate pseudo-pairing of separately generated scRNA-seq and scATAC-seq profiles. Unlike approaches primarily optimized for cross-modality reconstruction or latent alignment, scLAP explicitly targets reciprocal cell retrieval and one-to-one assignment. On prespecified cell-level partitions of a paired-liver reference, scLAP preserved exact-cell and sample-level structure. It also showed higher retrieval than a validation-selected native-peak scButterfly configuration and the study-specific Con-AAE and scMODAL adaptations on the fixed test partition. The leave-one-sample-out analysis nevertheless showed that exact-pair retrieval decreased substantially for unseen samples, delimiting the model’s current generalization. Applied to independent HCC datasets, the frozen model yielded 4007 similarity-filtered candidate RNA-ATAC pseudo-pairs spanning malignant, immune, and stromal compartments for exploratory downstream analysis.

The selected candidates showed biologically interpretable patterns in the two modalities, although these observations do not independently validate cell-level correspondence. Reanalysis of the original RNA count matrices recovered expected HCC and microenvironmental states. Separately, transferred-label ATAC analyses identified AP-1-associated malignant motifs, RUNX/ETS-associated T-cell motifs, SPIB/PU.1-associated macrophage motifs, and KLF/SP/RFX-associated fibroblast motifs. Fragment-library-blocked pseudobulk analysis identified an NRCAM-associated malignant accessibility peak. MET- and CAV1-proximal marker peaks, AP-1 enrichment, and co-accessibility are reported as exploratory observations rather than a validated unified regulatory mechanism.

Several limitations should be noted. First, six leave-one-sample-out folds produced a macro-mean Top 10 exact-pair retrieval rate of 5.23% and mean FOSCTTM of 0.2278, substantially weaker than the cell-level split and indicating limited exact-cell transfer to unseen samples. The paired reference contained only six source samples, limiting its coverage of inter-patient, disease-state, and technical heterogeneity. The feature basis remained transductive because the HVG list, peak universe, IDF values, motif scaling, and LSI projection were defined from the full paired reference, although model-input scaling was fitted on training cells in each fold. Second, on the untouched fixed test set, validation-calibrated q75 filtering achieved 4.57% exact-pair precision at 71.72% coverage; even the q99 threshold achieved 16.35% precision at only 3.21% coverage. The unpaired matches should therefore be regarded as similarity-filtered candidates, with pairing error addressed through aggregate, library-aware analyses rather than assumed absent. Third, pooled RNA cohorts and ATAC libraries differed in composition and technical processing, and cohort-separated matching confirmed cohort-dependent candidate competition. Finally, RNA-transferred ATAC labels are not independent ground truth, and fragment libraries have not been established as independent biological replicates. The RNA-label-blind GeneScore check detected above-null broad-lineage concordance but did not validate exact-cell identity. Future work will expand paired liver and HCC multiome references across independent patients and disease states, isolate feature construction within training folds, and optimize the model using broader cross-sample validation together with independent ATAC annotation and orthogonal biological validation.

## 4. Materials and Methods

### 4.1. Overall Framework

scLAP is a liver-adapted shared-latent model that learns aligned RNA–ATAC representations from co-assayed data and constructs model-guided pseudo-pairs for unpaired single-cell datasets. For a co-assayed training cell *i*, the model takes an ATAC feature vector *x_i_* ∈ ℝ^697^ and an RNA vector *y_i_* ∈ ℝ^2000^. Paired cell identities define positive ATAC–RNA pairs during training, whereas no cell-pair labels are required when applying the trained model to unpaired scRNA-seq and scATAC-seq datasets.

The framework comprises an ATAC encoder *E_A_*, an RNA encoder *E_R_*, modality-specific projection heads *P_A_* and *P_R_*, an RNA decoder *D_R_*, and an ATAC decoder *D_A_*. Given *x_i_* and *y_i_*, the encoders map the two modalities into hidden representations:

$$h_{i}^{A}=E_{A}\left(x_{i}\right),\;\;h_{i}^{R}=E_{R}\left(y_{i}\right),$$

which are subsequently projected into a unit-normalized shared latent space:

$$z_{i}^{A}=\frac{P_{A}\left(h_{i}^{A}\right)}{∥P_{A}\left(h_{i}^{A}\right){∥}_{2}},\;\;z_{i}^{R}=\frac{P_{R}\left(h_{i}^{R}\right)}{∥P_{R}\left(h_{i}^{R}\right){∥}_{2}}.$$

ATAC-to-RNA translation is defined as: ${\hat{y}}_{i}^{A}=D_{R}\left(h_{i}^{A}\right)$. During parent-model training, the RNA autoencoding pathway ${\hat{y}}_{i}^{R}=D_{R}\left(h_{i}^{R}\right)$, the ATAC autoencoding pathway ${\hat{x}}_{i}^{A}=D_{A}\left(h_{i}^{A}\right)$, and the reverse RNA-to-ATAC pathway ${\hat{x}}_{i}^{R}=D_{A}\left(h_{i}^{R}\right)$ were jointly optimized as auxiliary reconstruction pathways. These pathways helped preserve modality-specific information and mitigate the risk of latent collapse. The final scLAP model was initialized from the parent checkpoint and fine-tuned with *E_R_*, *P_R_*, *D_R_*, and *D_A_* frozen. Consequently, RNA reconstruction and RNA-to-ATAC translation did not contribute gradients to trainable parameters during final fine-tuning, whereas ATAC reconstruction continued to constrain the trainable ATAC encoder through the fixed ATAC decoder.

In the selected fine-tuning stage, the RNA-side projection head was frozen, whereas the ATAC-side projection head remained trainable. This distinction is used throughout the model and ablation descriptions.

### 4.2. Dataset and Feature Construction

The supervised paired reference was obtained from GSE223843, a single-cell multiomic liver dataset with shared RNA and ATAC cell identifiers. The split unit was the individual paired cell, stratified by sample prefix with random seed 42, yielding 15,095 training, 3234 validation, and 3235 test cells. The train/validation/test counts were 848/182/181 for Ctrl, 7161/1534/1535 for Ctrl3, 1577/338/338 for Fontan1, 2262/485/484 for Fontan2, 2634/564/565 for Fontan3, and 613/131/132 for Fontan4. Thus, all six samples occurred in every partition, and the reported benchmark is not a donor- or sample-level holdout. The 2000-gene list, consensus peak universe, TF-IDF/LSI basis, and motif-feature scaling were constructed from the full paired reference before cell splitting, whereas the 697 model-input feature means and standard deviations were estimated from training cells. The reported results therefore use transductive fixed-reference feature construction and should be interpreted as cell-level benchmarking within that reference. RNA input features consisted of the 2000 highly variable genes selected from the paired reference. ATAC features were constructed from a common 500-bp consensus peak space. Peak accessibility was transformed using TF-IDF, and the first 50 LSI components were retained:

For the leave-one-sample-out analysis, each of Ctrl, Ctrl3, Fontan1, Fontan2, Fontan3, and Fontan4 was excluded in turn from all model fitting and checkpoint selection. The remaining five samples were split into training and validation cells using seed 42, and the complete parent-training, RNA-anchored fine-tuning, and selected final scLAP fine-tuning sequence was repeated from random initialization. ATAC model-input means and standard deviations were estimated from the training cells in each fold. The fixed 2000-gene list and TF/LSI feature basis were retained to match the submitted model, so this analysis tested generalization of the learned mapping under the transductive reference feature definition rather than fully refitting the feature basis in every fold. Retrieval was evaluated only among paired RNA and ATAC cells from the unseen sample. Across-sample 95% confidence intervals were calculated from the six held-out-sample estimates using the t distribution.

$${\mathrm{tf}}_{\mathrm{pc}}=\frac{C_{\mathrm{pc}}}{\sum_{p^{\prime }}C_{p^{\prime }c}},\;\;{\mathrm{idf}}_{p}=\log\left(1+\frac{N}{1+\sum_{c}1\left(C_{\mathrm{pc}}>0\right)}\right),$$

*V_pc_* = log(1 + 10,000 × tf*_pc_*idf*_p_*).

Human JASPAR2022 [30] CORE PWMs were scanned against hg38 consensus peak sequences using motifmatchr after duplicated motif names were removed. Transcription factor motif activity was computed as:

*A*^TF^ = *VM*,

where *V* is the cell-by-peak TF-IDF matrix and M is the peak-by-motif matrix. Transcription factor-wise z-scoring was applied across cells. The final ATAC input concatenated 647 transcription factor motif activity features and 50 LSI features, producing a 697-dimensional vector per cell. For external fragment-derived scATAC-seq data, fragments were counted in the fixed paired-reference peak space; missing peaks contributed zeros, and the paired-reference IDF vector, motif matrix, and LSI loadings were reused without fitting independent query LSI axes. Motif activity was z-scored within each external ATAC cohort before concatenation with the projected LSI coordinates.

### 4.3. Model Architecture and Training Objective

Both modality encoders used residual multilayer perceptrons with GELU activation, layer normalization, and dropout. A residual block was defined as:

RB(*u*) = *u* + Dropout[*W*_2_ Dropout(*ϕ*(*W*_1_ LN(*u*)))],

where LN denotes layer normalization, and *ϕ* is the GELU activation function. The selected model was initialized from the parent checkpoint and fine-tuned with the RNA encoder, RNA projection head, RNA decoder, and ATAC decoder frozen, leaving the ATAC-side alignment components trainable. For a mini-batch of B paired cells, the ATAC-to-RNA translation loss was defined as:

$$L_{\mathrm{trans}}=0.35\;L_{\mathrm{SmoothL}1}\left({\hat{Y}}^{A},Y\right)+0.20\;\left[1-\frac{1}{B}\sum_{i=1}^{B}r\left({\hat{y}}_{i}^{A},y_{i}\right)\right].$$

Here, Smooth L1 denotes the robust regression loss between predicted and observed RNA profiles, and *r*(⋅) is Pearson correlation across genes within each cell.

Because the selected fine-tuning run froze the RNA encoder, RNA projection head, RNA decoder, and ATAC decoder, RNA reconstruction and RNA-to-ATAC translation did not contribute gradients to trainable parameters and were omitted from the effective final objective. ATAC reconstruction was retained as the only active reconstruction constraint because its gradient propagated through the fixed ATAC decoder to the trainable ATAC encoder:

$$L_{\mathrm{rec}}=0.03\;L_{\mathrm{ATACrec}}=0.03\;L_{\mathrm{SmoothL}1}\left({\hat{X}}^{A},X\right).$$

Matched RNA and ATAC embeddings were aligned using cosine similarity:

$$L_{\mathrm{paircos}}=1-\frac{1}{B}\sum_{i=1}^{B}{\left(z_{i}^{A}\right)}^{⊤}z_{i}^{R}.$$

To optimize cell retrieval, scLAP used a symmetric InfoNCE loss:

$$L_{\mathrm{InfoNCE}}=-\frac{1}{2B}\sum_{i=1}^{B}\left[\log\frac{\exp\left(S_{\mathrm{ii}}\right)}{\sum_{j}\exp\left(S_{\mathrm{ij}}\right)}+\log\frac{\exp\left(S_{\mathrm{ii}}\right)}{\sum_{j}\exp\left(S_{\mathrm{ji}}\right)}\right].$$

The temperature was set to *τ* = 0.03, and the mini-batch similarity logits were defined as $S_{\mathrm{ij}}={\cos(z}_{i}^{A},z_{j}^{R})/\tau$. This loss promoted reciprocal ATAC–RNA retrieval within each batch. To further separate highly similar but non-matched cells, we added hard-negative margin losses:

$$L_{\mathrm{HN}}=\frac{1}{2B}\sum_{i}\left[\max\left(0,m+\max_{j\neq i}S_{\mathrm{ij}}-S_{\mathrm{ii}}\right)+\max\left(0,m+\max_{j\neq i}S_{\mathrm{ji}}-S_{\mathrm{ii}}\right)\right].$$

A same-sample hard-negative term used the same formulation but restricted the negative pool to non-matched cells from the same sample prefix. To reduce many-to-one hub matching, a balanced batch loss was introduced to penalize unequal softmax assignment mass:

$$L_{\mathrm{balance}}=\frac{1}{B}\sum_{j}{\left(\sum_{i}P_{\mathrm{ij}}^{\mathrm{row}}-1\right)}^{2}+\frac{1}{B}\sum_{i}{\left(\sum_{j}P_{\mathrm{ij}}^{\mathrm{col}}-1\right)}^{2}$$

$$P_{\mathrm{ij}}^{\mathrm{row}}=\frac{\exp\left(S_{\mathrm{ij}}\right)}{\sum_{k}\exp\left(S_{\mathrm{ik}}\right)},\;\;P_{\mathrm{ij}}^{\mathrm{col}}=\frac{\exp\left(S_{\mathrm{ij}}\right)}{\sum_{k}\exp\left(S_{\mathrm{kj}}\right)}.$$

The Sinkhorn identity loss exponentiated *S*, performed five alternating row and column normalizations with epsilon = 1.0, and maximized diagonal assignment probability:

$$L_{\mathrm{Sinkhorn}}=-\frac{1}{B}\sum_{i=1}^{B}\log Q_{\mathrm{ii}},$$

where *Q* represents the doubly normalized mini-batch assignment matrix. A VICReg-style term was applied to hidden representations:

ℒ_VICReg_ = 0.01 ℒ_inv_ + 0.02 ℒ_var_ + 0.001 ℒ_cov_.

Finally, a cycle-consistency term re-encoded the RNA predicted from ATAC and encouraged it to remain close to the original ATAC latent vector:

$$L_{\mathrm{cycle}}=1-\frac{1}{B}\sum_{i=1}^{B}{\left(z_{i}^{\hat{Y}}\right)}^{⊤}z_{i}^{A},\;\;z_{i}^{\hat{Y}}=\frac{P_{R}\left(E_{R}\left({\hat{y}}_{i}^{A}\right)\right)}{∥P_{R}\left(E_{R}\left({\hat{y}}_{i}^{A}\right)\right){∥}_{2}}.$$

The effective final scLAP objective was therefore:

$$\begin{array}{cc} L= & L_{\mathrm{trans}}+L_{\mathrm{rec}}+0.60\;L_{\mathrm{paircos}}+2.00\;L_{\mathrm{InfoNCE}}\\ & +0.25\;L_{\mathrm{HN}}+0.12\;L_{\mathrm{sameSampleHN}}+0.20\;L_{\mathrm{balance}}+0.10\;L_{\mathrm{Sinkhorn}}\\ & +L_{\mathrm{VICReg}}+0.05\;L_{\mathrm{cycle}}. \end{array}$$

The selected model used a hidden width of 1024, 512-dimensional encoder representations, 128-dimensional normalized latent vectors, four encoder residual blocks, two decoder residual blocks, GELU activation, and dropout of 0.12. Fine-tuning used AdamW (learning rate 5 × 10^−5^; weight decay 1 × 10^−4^), a cosine-annealing schedule over at most 50 epochs, batch size 2048, mixed-precision training, and gradient-norm clipping at 5. Model selection used the validation score combining bidirectional Top 1/Top 10 retrieval, bidirectional FOSCTTM, and ATAC-to-RNA cell-wise Pearson correlation; early stopping patience was 10 epochs and the random seed was 42.

Training proceeded in three stages. Stage 1 comprised 30 epochs of RNA autoencoder warm-up followed by joint training for at most 90 epochs (learning rate, 3 × 10^−4^; dropout, 0.15; temperature, 0.05; hard-negative margin, 0.10; patience, 16). Stage 2 initialized from the Stage 1 checkpoint and applied RNA-anchored fine-tuning for at most 70 epochs (learning rate, 1 × 10^−4^; dropout, 0.15; temperature, 0.03; margin, 0.15; patience, 12). Stage 3 initialized from the Stage 2 checkpoint and used the selected final objective for at most 50 epochs. The complete stage-specific loss weights and checkpoint identifiers are provided in Supplementary Tables S8 and S9. The SHA-256 digest of the final checkpoint was a0511044eb6a46aa22119bfc822026b4cd5cb23116548332d2a4a1e98904c23b.

For comparison, Con-AAE used 1000 training-selected binary peaks and a 50-dimensional latent space for 30 epochs; scMODAL used the linked-feature interface with the 697 ATAC features and 2000 RNA features for 2000 training steps. The revised scButterfly benchmark used the submitted 2000 log-normalized RNA genes and 20,000 training-selected binary peaks transformed by training-derived TF-IDF. Its RNA and ATAC autoencoders were pretrained for at most 100 epochs each, followed by at most 200 integration epochs with patience 30. Learning rates of 1 × 10^−3^ and 3 × 10^−4^ were prespecified, and the final learning rate was selected using only validation retrieval. Test metrics were calculated once for the selected run. A ridge regression control mapped the 697 standardized ATAC inputs to the 2000 row-standardized RNA outputs; the penalty was selected from 0.1 to 10,000 using only the validation partition.

### 4.4. Pseudo-Pairing of Unpaired scRNA-Seq and scATAC-Seq Cells

ATAC-to-RNA translation performance was evaluated by R^2^, cell-wise Pearson correlation, and gene-wise Pearson correlation. Cell-level alignment was evaluated by Top 1, Top 5, Top 10, and Top 50 exact-cell retrieval rates, median rank, fraction of samples closer than the true match (FOSCTTM), and mutual-nearest-neighbor statistics in both ATAC-to-RNA and RNA-to-ATAC directions. R^2^ and FOSCTTM were defined as:

$$R^{2}=1-\frac{\sum_{i,g}{\left({\hat{y}}_{\mathrm{ig}}-y_{\mathrm{ig}}\right)}^{2}}{\sum_{i,g}{\left(y_{\mathrm{ig}}-\overset{-}{y}\right)}^{2}},\;\;{\mathrm{FOSCTTM}}_{i}=\frac{1}{N-1}\sum_{j\neq i}1\left[s_{\mathrm{ij}}>s_{\mathrm{ii}}\right].$$

For each query cell, FOSCTTM was calculated as the number of non-matched candidates with a higher latent cosine similarity than the true paired reference, divided by the total number of non-matched candidates. We reported the mean across query cells. FOSCTTM therefore summarizes ranking quality and is not an exact-pair accuracy measure. Sample-stratified values were reported for the fixed cell-level benchmark, and uncertainty across unseen samples was estimated from the six leave-one-sample-out folds.

For unpaired pseudo-pairing, let *A* and *R* denote the ATAC and RNA cell sets, respectively. The cosine similarity between ATAC cell *a* and RNA cell *r* was defined as:

$$s\left(a,r\right)={\cos(z}_{a}^{A},z_{r}^{R}).$$

Candidate ATAC-RNA pairs were retained if they satisfied mutual Top *K* retrieval and a cosine-similarity threshold:

*E_K_* = {(*a*,*r*):*a* ∈ *A*, *r* ∈ *R*, *r* ∈ *Top K_R_*(*a*), *a* ∈ *Top K_A_*(*r*), *s*(*a*,*r*) ≥ *θ*},

where *K* = 10 and the corrected threshold was theta = 0.815683. Retained candidate pairs were then sorted by decreasing similarity, followed by greedy one-to-one assignment.

The corrected threshold, 0.815683, was the 75th percentile of true-pair cosine similarities in the co-assay validation partition and was locked before evaluation on the untouched fixed test partition. Precision-coverage sensitivity was evaluated at validation quantiles q50, q60, q70, q75, q80, q90, q95, q97.5, and q99. Applying the corrected q75 threshold to the external HCC embeddings retained 4007 of the 4008 candidates obtained with the previous test-derived threshold of 0.815542; the one excluded RNA anchor was unclassified and did not enter the annotated ATAC comparisons.

### 4.5. RNA Annotation Harmonization and Cohort Summaries

Dataset-provided RNA annotations were harmonized by collapsing only spelling and plurality variants into eight categories: Malignant, T cell, TAM, CAF, TEC, HPC-like, B cell, and unclassified. Specifically, B cell/B cells/B-cell, T cell/T cells/T-cell, Malignant cell/Malignant cells, CAF/CAFs, TAM/TAMs, and TEC/TECs were mapped to common labels; HPC-like was present only in GSE125449. No de novo reassignment was performed. Records labeled unclassified in the source metadata remained unclassified and were retained in pairing summaries but excluded from cell-type differential accessibility tests. Candidate retention was summarized against the full input RNA pool by cohort and harmonized cell type.

To assess cohort sensitivity, the complete mutual Top 10 and greedy assignment procedure was repeated separately for each RNA cohort against the same GSE227265 ATAC pool, using the locked validation-derived q75 threshold. Separate-cohort selections were compared with the pooled candidate set by exact RNA-ATAC pair identity and summarized by harmonized RNA cell type and ATAC barcode-library index.

For the RNA-label-blind ATAC marker check, six broad-lineage panels were prespecified: T-cell, myeloid, B-cell, fibroblast, endothelial, and epithelial/hepatobiliary. GeneScore values were log transformed and standardized by gene across the 3373 annotated ATAC candidates; panel scores were the mean standardized values of available genes, and the maximum panel score defined the ATAC marker class. RNA labels were used only after scoring to calculate concordance. Significance was assessed with 2000 permutations of the RNA broad-lineage labels within ATAC fragment-library strata.

### 4.6. Fragment-Library-Blocked Differential Accessibility

For each transferred RNA cell type, ATAC peak counts were aggregated within fragment library to create target and pooled-other pseudobulks. A library was eligible when it contained at least 10 target cells and 20 other labeled cells, and a cell type was tested only when at least three libraries were eligible. Peaks were retained when they had at least 10 counts in at least three pseudobulk samples. edgeR models used TMM normalization, robust dispersion estimation, and a quasi-likelihood design of ~FragmentLibrary + group. The group target coefficient compared the target cell type with pooled other labeled groups while blocking for fragment library. Peaks with FDR ≤ 0.05 and |log2 fold change| ≥ 1 were called significant. Fragment libraries were treated as blocking units and were not assumed to be confirmed independent biological donors.

For the NRCAM-associated peak at chr7:108358443–108358943, raw counts and library-size-normalized counts per million were tabulated separately for malignant and pooled-other pseudobulks in each eligible fragment library. Robustness was assessed by refitting the same edgeR quasi-likelihood model nine times, excluding one eligible library per fit. We recorded the log2 fold change, nominal P value, and FDR for the NRCAM-associated peak in every leave-one-library-out analysis.

## Figures and Tables

**Table 1:** Fixed-split cross-modal retrieval performance of scLAP and comparison methods on paired liver co-assay data.

Method	Evaluation Set	Cells (n)	Top 1 (%)	Top 10 (%)	Top 50 (%)	Mean FOSCTTM	Cell-Wise Pearson
scLAP	Validation	3234	4.21	21.89	49.20	0.0438	0.6650
scLAP	Test	3235	4.82	21.48	50.14	0.0431	0.6631
Con-AAE	Test	3235	0.22	1.64	6.12	0.3431	0.5255
scButterfly (native peaks)	Test	3235	1.21	8.93	27.39	0.0811	0.6563
scMODAL	Test	3235	0.03	0.68	3.15	0.3770	NA

## Data Availability

The original contributions presented in this study are included in the article/Supplementary Materials. Further inquiries can be directed to the corresponding authors.

## Supplementary Materials

The following supporting information can be downloaded at https://www.mdpi.com/article/10.3390/ijms27219888/s1.

## Abbreviations

scRNA-seq	single-cell RNA sequencing
scATAC-seq	single-cell assay for transposase-accessible chromatin sequencing
scLAP	single-cell Liver-adapted Alignment and Pseudo-pairing
FOSCTTM	fraction of samples closer than the true match (FOSCTTM
PLC	Primary liver cancer
HCC	Hepatocellular carcinoma
LSI	latent semantic indexing
TECs	tumor endothelial cells
TAMs	tumor-associated macrophages
