# Supplementary material for: scLAP: A Liver-Adapted Contrastive Shared-Latent Framework for Pseudo-Pairing Single-Cell Transcriptomes and Chromatin Accessibility Profiles
Source: Int J Mol Sci. 2026 Nov 4;27(21):9888. doi: 10.3390/ijms27219888 (PMC13764276; doi:10.3390/ijms27219888)
Supplement: Supplementary file 1 [file ijms-27-09888-s001.zip › ijms-4659574-supplementary.pdf]

Supplementary Tables

Supplementary Table S1

Leave-one-sample-out performance after complete fold-specific three-stage training. Filtered precision and coverage use validation q75, mutual Top10, and greedy one-to-one assignment.

Held-out sample	n	Top1 (%)	Top10 (%)	Mean FOSCTTM	Cell-wise Pearson	Filtered precision (%)	Filtered coverage (%)
Ctrl	1,211	1.90	9.58	0.2009	0.5808	3.10	50.62
Ctrl3	10,230	0.11	0.80	0.3144	0.4627	0.08	23.86
Fontan1	2,253	0.44	3.73	0.2530	0.6779	1.07	41.37
Fontan2	3,231	0.74	3.74	0.1890	0.6948	0.26	23.52
Fontan3	3,763	0.72	5.53	0.1673	0.5720	1.33	38.00
Fontan4	876	1.48	7.99	0.2423	0.7368	2.07	44.06

Supplementary Table S2

Precision-coverage sensitivity of the complete pairing procedure on the untouched fixed test partition. Every threshold was derived from true-pair similarities in the fixed validation partition.

Validation quantile	Cosine threshold	Selected pairs	Exact pairs	Precision (%)	Exact recall (%)	Coverage (%)	Same-sample pairs (%)
q50	0.767734	2,651	114	4.30	3.52	81.95	95.62
q60	0.786801	2,631	114	4.33	3.52	81.33	95.74
q70	0.805729	2,511	109	4.34	3.37	77.62	95.94
q75	0.815683	2,320	106	4.57	3.28	71.72	96.12
q80	0.824548	2,043	100	4.89	3.09	63.15	96.23
q90	0.848186	1,205	77	6.39	2.38	37.25	96.02
q95	0.868870	538	56	10.41	1.73	16.63	95.72
q97.5	0.886383	224	33	14.73	1.02	6.92	94.64
q99	0.902571	104	17	16.35	0.53	3.21	94.23

Supplementary Table S3

RNA-cohort sensitivity of pooled and cohort-separated HCC matching using validation q75, mutual Top10, and greedy one-to-one assignment.

RNA cohort	Eligible RNA	Pooled pairs	Pooled selection (%)	Pooled composition enrichment	Separate-cohort pairs	Separate selection of RNA (%)	Pair overlap with pooled	Overlap of separate set (%)
GSE125449	9,946	419	4.21	1.88	1,741	17.50	231	13.27
GSE151530	56,721	2,514	4.43	1.98	3,507	6.18	2,216	63.19
GSE189903	112,506	1,074	0.95	0.43	2,101	1.87	895	42.60

Supplementary Table S4

Leave-one-fragment-library-out edgeR sensitivity for the intronic NRCAM-associated peak chr7:108358443-108358943. The row labelled NA is the full nine-library model.

Excluded library	Retained libraries	log2FC	logCPM	F	P value	FDR	Positive direction	FDR < 0.05
NA	9	4.8848	6.4600	29.0272	7.148e-08	1.256e-03	Yes	Yes
Lib_1	8	5.3215	6.3705	26.3362	2.873e-07	5.001e-03	Yes	Yes
Lib_12	8	5.0081	6.5969	31.2340	2.292e-08	3.788e-04	Yes	Yes
Lib_14	8	4.8086	6.5019	26.2822	2.955e-07	5.085e-03	Yes	Yes
Lib_15	8	5.0254	6.7578	34.2978	4.744e-09	6.495e-05	Yes	Yes
Lib_20	8	4.2527	5.9990	16.5288	4.795e-05	8.244e-01	Yes	No
Lib_3	8	5.5372	6.4563	31.1637	2.377e-08	4.169e-04	Yes	Yes
Lib_4	8	4.7834	6.5117	24.4944	7.464e-07	1.134e-02	Yes	Yes
Lib_5	8	4.3379	6.5376	18.4353	1.759e-05	2.780e-01	Yes	No
Lib_8	8	5.0187	6.7452	29.8747	4.620e-08	6.832e-04	Yes	Yes

Supplementary Table S5

ATAC barcode-library representation in each RNA-cohort-separated match. Library indices are parsed from the GSE227265 aggregated barcode suffixes and are not assumed to represent independent biological donors.

RNA cohort	ATAC library index	Input ATAC cells	Selected pairs	Selection within library (%)	Composition enrichment
GSE125449	1	5,820	103	1.77	0.56
GSE125449	10	305	11	3.61	1.15
GSE125449	11	323	8	2.48	0.79
GSE125449	12	3,659	150	4.10	1.30
GSE125449	13	471	22	4.67	1.49
GSE125449	14	3,899	89	2.28	0.73
GSE125449	15	2,134	130	6.09	1.94
GSE125449	16	911	30	3.29	1.05
GSE125449	17	166	18	10.84	3.45
GSE125449	18	174	7	4.02	1.28
GSE125449	19	516	17	3.29	1.05
GSE125449	2	4,483	8	0.18	0.06
GSE125449	20	5,428	169	3.11	0.99
GSE125449	3	5,329	51	0.96	0.30
GSE125449	4	5,065	292	5.77	1.84
GSE125449	5	11,354	370	3.26	1.04
GSE125449	6	779	34	4.36	1.39
GSE125449	7	908	33	3.63	1.16
GSE125449	8	3,396	186	5.48	1.74
GSE125449	9	301	13	4.32	1.37
GSE151530	1	5,820	200	3.44	0.54
GSE151530	10	305	19	6.23	0.98
GSE151530	11	323	21	6.50	1.03
GSE151530	12	3,659	266	7.27	1.15
GSE151530	13	471	41	8.70	1.38

RNA cohort	ATAC library index	Input ATAC cells	Selected pairs	Selection within library (%)	Composition enrichment
GSE151530	14	3,899	199	5.10	0.81
GSE151530	15	2,134	235	11.01	1.74
GSE151530	16	911	63	6.92	1.09
GSE151530	17	166	26	15.66	2.48
GSE151530	18	174	10	5.75	0.91
GSE151530	19	516	25	4.84	0.77
GSE151530	2	4,483	46	1.03	0.16
GSE151530	20	5,428	473	8.71	1.38
GSE151530	3	5,329	93	1.75	0.28
GSE151530	4	5,065	439	8.67	1.37
GSE151530	5	11,354	838	7.38	1.17
GSE151530	6	779	68	8.73	1.38
GSE151530	7	908	56	6.17	0.97
GSE151530	8	3,396	358	10.54	1.67
GSE151530	9	301	31	10.30	1.63
GSE189903	1	5,820	72	1.24	0.33
GSE189903	10	305	18	5.90	1.56
GSE189903	11	323	13	4.02	1.06
GSE189903	12	3,659	191	5.22	1.38
GSE189903	13	471	42	8.92	2.35
GSE189903	14	3,899	117	3.00	0.79
GSE189903	15	2,134	217	10.17	2.68
GSE189903	16	911	67	7.35	1.94
GSE189903	17	166	25	15.06	3.97
GSE189903	18	174	8	4.60	1.21
GSE189903	19	516	26	5.04	1.33
GSE189903	2	4,483	3	0.07	0.02
GSE189903	20	5,428	214	3.94	1.04

RNA cohort	ATAC library index	Input ATAC cells	Selected pairs	Selection within library (%)	Composition enrichment
GSE189903	3	5,329	18	0.34	0.09
GSE189903	4	5,065	212	4.19	1.10
GSE189903	5	11,354	488	4.30	1.13
GSE189903	6	779	86	11.04	2.91
GSE189903	7	908	68	7.49	1.98
GSE189903	8	3,396	199	5.86	1.55
GSE189903	9	301	17	5.65	1.49

Supplementary Table S6

Validation-selected native-peak scButterfly and supervised ridge controls on the fixed test partition. Latent-space and predicted-expression-space retrieval are reported separately and are not directly interchangeable.

Configuration	Space	n	Top1 (%)	Top10 (%)	Top50 (%)	Mean FOSCTTM	Cell-wise Pearson
scLAP shared latent	Latent	3235	4.82	21.48	50.14	0.0431	0.6631
scLAP predicted expression	Predicted expression	3235	0.46	4.48	16.72	0.1339	0.6631
Ridge predicted expression	Predicted expression	3235	0.25	3.96	13.04	0.1817	0.6569
scButterfly predicted expression	Predicted expression	3235	0.43	3.59	12.15	0.1780	0.6563
scButterfly native-peak latent	Latent	3235	1.21	8.93	27.39	0.0811	0.6563

Supplementary Table S7

RNA-label-blind ATAC GeneScore marker-concordance check. Overall concordance was 30.00% among 3,373 annotated candidates, compared with a library-conditioned permutation mean of 22.16% (empirical P = 0.00050). This broad-lineage check is not exact-cell ground truth.

Broad lineage	n	Concordant n	Concordance
T_cell	919	322	35.04%
Myeloid	512	142	27.73%
B_cell	44	11	25.00%
Fibroblast	210	54	25.71%

Broad lineage	n	Concordant n	Concordance
Endothelial	167	67	40.12%
Epithelial_hepatobiliary	1521	416	27.35%

Supplementary Table S8

Three-stage scLAP training configuration. All stages used hidden width 1,024, hidden representation 512, latent dimension 128, four encoder residual blocks, two decoder residual blocks, GELU activation, batch size 2,048, AdamW weight decay 1 × 10−4, cosine annealing, gradient clipping at 5, and seed 42.

Stage	Run	Maximum epochs	RNA warm-up	Learning rate	Dropout	Temperature	Margin	Patience	Frozen modules	Checkpoint SHA-256
1	preRNA30_b2048_finetune	90	30	3.0e-04	0.15	0.05	0.1	16	None	4b46a0f590dff12a…
2	anchorRNA_matchstrong	70	0	1.0e-04	0.15	0.03	0.15	12	RNA encoder, RNA projection, RNA decoder, ATAC decoder	1a962b6ade71aaff…
3	eval_anchor_strict_v2	50	0	5.0e-05	0.12	0.03	0.18	10	RNA encoder, RNA projection, RNA decoder, ATAC decoder	a0511044eb6a46aa…

Supplementary Table S9

Stage-specific objective weights. In Stages 2 and 3, terms whose computational paths were fully frozen did not update trainable parameters; the effective final objective is defined in Section 4.3.

Loss weight	Stage 1	Stage 2	Stage 3
w_trans	0.7	0.4	0.35
w_trans_corr	0.3	0.2	0.2
w_rna_recon	0.25	0.05	0.03
w_rna_recon_corr	0.1	0.02	0.01
w_atac_recon	0.1	0.05	0.03
w_rna_to_atac	0.05	0.02	0.01
w_pair_cos	0.2	0.5	0.6
w_infonce	0.8	1.5	2
w_hardneg	0.1	0.2	0.25
w_same_sample_hardneg	0.05	0.1	0.12

Loss weight	Stage 1	Stage 2	Stage 3
w_balance	0.1	0.15	0.2
w_sinkhorn	0.05	0.08	0.1
w_vicreg_inv	0.03	0.01	0.01
w_vicreg_var	0.03	0.02	0.02
w_vicreg_cov	0.003	0.001	0.001
w_cycle	0.05	0.05	0.05
